# Relationships between Follicle-Stimulating Hormone and Adiponectin in Postmenopausal Women

**DOI:** 10.3390/metabo10100420

**Published:** 2020-10-19

**Authors:** Wan-Yu Huang, Dar-Ren Chen, Chew-Teng Kor, Ting-Yu Chen, Po-Te Lin, Joseph Ta Chien Tseng, Hung-Ming Wu

**Affiliations:** 1 Pediatrics of Kung-Ten General Hospital, Taichung City 433, Taiwan; juliahuang01@gmail.com; 2 Comprehensive Breast Cancer Center, Changhua Christian Hospital, Changhua 500, Taiwan; 115045@cch.org.tw; 3 Internal Medicine Research Center, Changhua Christian Hospital, Changhua 500, Taiwan; 179297@cch.org.tw; 4 Inflammation Research & Drug Development Center, Changhua Christian Hospital, Changhua 500, Taiwan; 180662@cch.org.tw (T.-Y.C.); 11816@cch.org.tw (P.-T.L.); 5 Department of Biotechnology and Bioindustry Sciences, National Cheng-Kung University, Tainan 701, Taiwan; tctseng@mail.ncku.edu.tw; 6 Department of Neurology, Changhua Christian Hospital, Changhua 500, Taiwan; 7 Graduate Institute of Acupuncture Science, China Medical University, Taichung 406, Taiwan

**Keywords:** FSH, adiponectin, insulin resistance, postmenopause, and breast cancer

## Abstract

Beyond fertility, follicle-stimulating hormone (FSH) may exert action on adipocytes, which are the major source of adiponectin and leptin, linking to insulin resistance. Therefore, we evaluated the relationships between FSH and adipocyte-derived hormones. This cross-sectional study enrolled postmenopausal women aged 40–65 years. The variables measured in this study included clinical parameters, fasting levels of sex hormones, glucose, insulin, and adipokines. A total of 261 women without breast cancer, 88 women with breast cancer receiving tamoxifen, and 59 women with breast cancer receiving additional gonadotropin-releasing hormone analogs were enrolled in this study. Significant differences in the levels of adiponectin, leptin, and FSH were observed between the non-breast cancer group and the breast cancer groups. Spearman’s rank test revealed significant associations of FSH with either body mass index (BMI) or homeostatic model assessment of insulin resistance (HOMA-IR) values in the non-breast cancer group. After adjusting for BMI, age, and menopause duration, FSH levels were significantly associated with adiponectin (*p* < 0.001) and the leptin-to-adiponectin ratio (*p* = 0.008) in the non-breast cancer group, but they were only significantly associated with adiponectin (*p* = 0.001) in the breast cancer group receiving tamoxifen. Our data show that FSH levels are independently associated with adiponectin levels in postmenopausal women, suggesting that adiponectin may link FSH to metabolic relationships in postmenopausal female.

## 1. Introduction

Follicle-stimulating hormone (FSH) exerts its effects in sexual organs (e.g., ovaries and testes) to stimulate ovarian follicle growth and maturation as well as 17β-estradiol (E2) secretion from granulosa cells in females [1]. However, FSH has been demonstrated to have biological activities on the FSH receptor in extra-gonadal tissues, such as bone, muscle, and fat [2,3,4]. This understanding leads to a rapid exploration of FSH biological actions in many biological systems in addition to the gonads over the past decade. 

FSH levels increase progressively with age, and a more distinct rise occurs during the menopause transition in most women [5]. Coincidently, an increased risk of metabolic and cardiovascular diseases is observed in women of this age. Although multiple factors, such as estrogen deprivation, contribute to this issue from a premenopausal to a postmenopausal state [6,7], increasing evidence shows that FSH might play a role in metabolic changes [8]. The findings from The Study of Women’s Health Across the Nation (SWAN) demonstrated that serum FSH level is associated with body weight gain and increased visceral adiposity [9]. Further studies reported that FSH is negatively associated with fasting glucose levels and glycated hemoglobin as well as with a higher prevalence of prediabetes and diabetes in postmenopausal women [10,11]. These results suggest that FSH might be associated with adiposity and insulin resistance. 

Adipose tissue is an active metabolic and endocrine organ that regulates various metabolic functions. This organ releases the adipocyte-derived hormones leptin, adiponectin, and resistin, which have important roles in metabolic pathological processes and insulin resistance [12,13]. However, few studies have evaluated the relationship between adipocyte-derived hormones and FSH in humans. Sowers et al. reported that adipokines, such as adiponectin and resistin, are significantly different between the menopause transition and the premenopausal stage [14]. Notably, increases in the FSH changes by menopausal stage are positively associated with higher adiponectin concentrations [14], suggesting that FSH might be linked to aspects of adipocyte-derived factors, metabolic disorder, and insulin resistance. In addition, adiponectin has been considered to represent the link factor in cancer such as breast cancer and colon cancer through different molecular mechanisms (e.g., anti-inflammation effects) [15,16]. Recent evidence further showed that peripheral adiponectin levels are associated with increased breast cancer risk [17].

Insulin resistance is generally considered to be a risk factor for cardiovascular disease. Adipocyte-derived hormone, in particular, a lower adiponectin level, is a significant risk factor for insulin resistance and the development of diabetes [13,18]. Therefore, this study determined the associations between FSH and adipocyte-derived hormones in postmenopausal women. Adjuvant tamoxifen therapy with/without gonadotropin-releasing hormone (GnRH) analogs is frequently used for the patient with breast cancer. However, little is known about whether the adipocyte-derived hormones are altered in breast cancer patients who receive the medication. Additionally, we evaluated the influence of adjuvant tamoxifen therapy with/without GnRH analogs for breast cancer on adipocyte-derived hormones concentrations and their relation with metabolic features.

## 2. Results 

### 2.1. Demographic, Clinic, and Laboratory Data

A total of 261 relatively healthy postmenopausal women (non-BrCa), 88 postmenopausal women with breast cancer receiving tamoxifen therapy (BrCa), and 59 women with breast cancer receiving both tamoxifen and a GnRH analog injection (BrCa-Gn) who fulfilled the entry criteria were enrolled in this study. Significant differences in the levels of circulating FSH, adiponectin, leptin, resistin, and the lipid profile, including total cholesterol, triglycerides, HLD-C, and LDL-C (Table 1) were observed between the non-BrCa group and the BrCa group, but no differences were detected in the number of postmenopausal years since the last menstrual period, blood pressure, BMI, or fasting glucose (Table 1). In addition, the BrCa group had higher levels of FSH (*p* < 0.001) and adiponectin (*p* = 0.048) than those in the BrCa-Gn group (Table 1). 

### 2.2. Correlation between FSH, BMI, and the HOMA-IR Values

**Figure 1:**
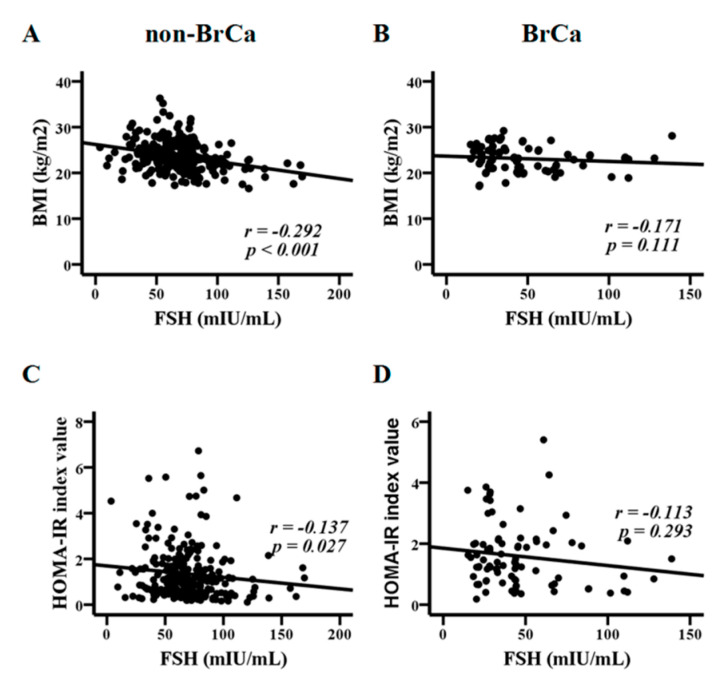
Associations between follicle-stimulating hormone (FSH), body mass index (BMI), and the homeostatic model assessment of insulin resistance (HOMA-IR) index values. FSH was correlated with BMI (**A**) and HOMA-IR (**C**) in the non-breast cancer (non-BrCa) group, but not with BMI (**B**) and HOMA-IR (**D**) in the breast cancer (BrCa) group. The statistical analysis was conducted with Spearman’s rank test.

**Figure 2:**
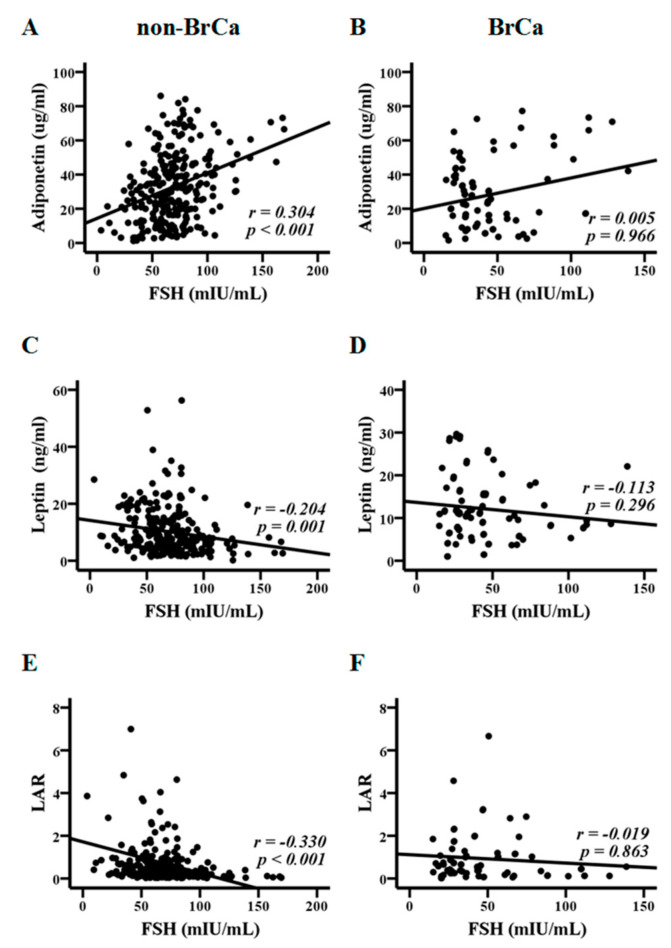
Associations between FSH, adiponectin, leptin, and the leptin to adiponectin ratio (LAR). FSH was correlated with adiponectin (**A**), leptin (**C**), and the LAR (**E**) in the non-breast cancer (non-BrCa) group, but not with adiponectin (**B**), leptin (**D**), and the LAR (**F**) in the breast cancer (BrCa) group. Statistical analysis was conducted by Spearman’s rank test.

FSH can affect total body weight and fat mass, particularly during menopause [9]. Spearman’s rank test revealed significant associations of FSH with BMI (*p* < 0.0001) and with HOMA-IR (*p* = 0.027) in the non-BrCa group, which were not observed in the BrCa group (Figure 1) and in the BrCa-Gn group. Significant associations of FSH with adiponectin (*p* < 0.0001), leptin (*p* = 0.001), and the leptin to adiponectin ratio (LAR) (*p* < 0.0001) were further revealed in the non-BrCa group (Figure 2A,C,E), but they were not significant with these three parameters in the BrCa group (Figure 2B,D,F). 

### 2.3. Multivariate Linear Regression Analyzes to Evaluate the Variables Associated with FSH

A multivariate linear regression model was used to examine the relationships between FSH and each of the adipocyte-derived hormones and LAR after logarithmic transformation for adiponectin, leptin, and LAR. When adjusting for BMI, age, and menopause duration, FSH levels were significantly associated with Log-adiponectin levels (*p* < 0.001) and Log-LAR (*p* < 0.001) in the non-BrCa group (Table 2).

## 3. Discussion

Investigating the associations between FSH and adipocyte-derived hormones provides insight into the role of FSH during metabolism in postmenopausal women. FSH was significantly associated with adiponectin levels in the non-BrCa group (*p* < 0.001) after adjusting for age, BMI, and menopause duration. These results indicate that adiponectin might be a link between FSH and metabolic disorders, such as insulin resistance and prediabetes, in postmenopausal women [10,19]. 

An increasing number of studies have characterized the relationship between FSH and body composition in humans, particularly in perimenopausal and postmenopausal women [20]. Positive correlations were observed between increased changes in FSH and the fat mass change at the six-year follow-up of a midlife study [21] and in infertile premenopausal women [22]. Another study reported an independent association between high FSH levels and low lean mass in younger postmenopausal women [23]. Several previous studies, such as the 11-year SWAN follow-up and the Pan Asia Menopause Study, showed that low serum levels of FSH occur in women with a high BMI [24,25,26,27,28,29]. These results seem paradoxical, but they probably arise from total body weight with different compositions of lean mass and fat mass along with different menopausal statuses (e.g., perimenopause, early and late postmenopause) among these women. Indeed, multiple factors are involved in the risk of gaining weight, including aging, lifestyle, genetics, and menopause-related symptoms (e.g., mood changes and sleep disturbances) [30,31,32]. In particular, estrogens and FSH are important factors that interact with body weight and body components during the menopausal transition [30]. In our relatively homogeneous postmenopausal population with very low levels of E2 and no history of chronic illness, our data show a negative association between serum FSH level and BMI, similar to previous findings [24,25,26,27,28,29]. Nevertheless, further studies are needed to provide evidence for the exact role of estrogens, FSH, and both body composition and body weight during menopause, as a recent animal study suggested that FSH has a detrimental effect on body composition [3]. Interestingly, the significant association between serum FSH and BMI was not observed in the BrCa and BrCa-Gn groups. One possibility is that tamoxifen treatment may affect gonadotropin release in the hypothalamic–pituitary axis [33], leading to a decrease in FSH levels in postmenopausal women, as observed in our BrCa and BrCa-Gn women (Table 1) and in the previous studies [33,34]. Another possibility is that tamoxifen, a selective estrogen receptor modulator, induces changes in body composition and glucose and lipid metabolism [35,36,37]. This finding is worthy of further investigation in patients undergoing tamoxifen therapy for breast cancer. 

Weight gain and fat composition changes are very common in aging women, particularly at menopause [32]. As adipose tissue is metabolically active and the main source of adipocytokines, including adiponectin and leptin in humans, the increased fat mass and fat redistribution (e.g., increased visceral fat) may alter genetic expression (e.g., adiponectin and leptin) in adipocytes [30]. Several studies have suggested a link between sex hormones and adiponectin metabolism [14,30,38,39]. FSH regulates lipid biosynthesis via three pathways in cultured preadipocytes [40,41] such as by enhancing peroxisome proliferator-activated receptor transcripts and increasing the expression of leptin and adiponectin, as observed in in vitro and in vivo studies [40,41]. Increases in the changes of FSH from the premenopausal to the postmenopausal stages are positively associated with adiponectin levels [14]. The present study showed that the FSH levels were positively associated with total cholesterol (*p* = 0.0265) and HDL cholesterol (*p* < 0.0001) as well as negatively with triglyceride levels (*p* = 0.0068). Furthermore, we found that the circulating FSH level was significantly associated with higher adiponectin levels, but not with leptin in relatively healthy postmenopausal women without breast cancer, after adjusting for age, BMI, and menopause duration. When blocking FSH release by a GnRH analog in hypothalamus for breast cancer patients, circulating adiponectin was further decreased compared with that in breast cancer patients receiving tamoxifen therapy only, suggesting that FSH has a role in adiponectin expression [40,41] in humans. In fact, the levels of FSH, adiponectin, and leptin were significantly different in the BrCa group from those in non-BrCa group. The observation implies that there is a possible role of adjuvant tamoxifen therapy on these hormone factors in breast-cancer patients. Due to the lack of a group with breast cancer without treatment in the present study, it could not exclude the possibility of alteration of adiponectin and leptin expression as the hidden risk factor involved in this phenomenon in these breast cancer women [42]. This is the first study to evaluate the relationships between FSH and adipocyte-derived hormones in a reasonably large (*n* = 408) sample of postmenopausal women. Adiponectin is the most consistent biochemical predictor of insulin resistance and diabetes with a dose–response relationship across diverse populations [18,43]. The LAR is a useful biomarker of adipose tissue dysfunction, indicating insulin resistance in non-diabetic subjects [13,44,45]. A significant correlation was observed between LAR and HOMA-IR in the non-BrCa (*r* = 0.396, *p* < 0.0001) and BrCa groups (*r* = 0.386, *p* = 0.0002), respectively. Further analysis showed that FSH was independently associated with the LAR in our postmenopausal women. Taken together, our results support that a higher FSH level was associated with lowering insulin resistance and diabetes in postmenopausal women [10,19]. 

## 4. Materials and Methods

### 4.1. Participants and Study Design

In this cross-sectional study, women (age 40–65 years) who visited the Changhua Christian Hospital between July 2017 and June 2019 were eligible. Postmenopause was defined as ≥12 consecutive months of amenorrhea prior to study entry. Gonadotropin-releasing hormone (GnRH) analogs can abolish sex hormone production (e.g., FSH) via inhibition of the pituitary-gonadal axis. The analogs are frequently used in women with breast cancer. The breast cancer cases receiving tamoxifen with/without GnRH could be as the treatment controls. Therefore, three subgroups of postmenopausal women were enrolled in this study: (I) postmenopausal women without breast cancer (non-BrCa), (II) postmenopausal women receiving adjuvant tamoxifen therapy for breast cancer for at least 1 year (BrCa), and (III) postmenopausal women receiving both tamoxifen and a gonadotropin-releasing hormone (GnRH) analog for breast cancer for at least 1 year (BrCa-Gn). Women were excluded if they were in the early or late perimenopausal stage, used hormone therapy, or had a history of chronic systemic diseases, including diabetes (defined as fasting glucose > 126 mg/mL, postprandial glucose > 200 mg/mL or on antidiabetic medication), hyperlipidemia (defined as total cholesterol > 240 mg/dL or triglycerides > 200 mg/dL or on statin medication), hypertension (defined as blood pressure >140/90 mmHg or on antihypertensive medication), or thyroid disease. Written informed consent was obtained from all subjects. This study was approved by the Changhua Christian Hospital Institutional Review Board (ID: CCH IRB No. 150609). 

### 4.2. Anthropometric Measures 

Blood specimens were collected from individuals in the morning after an overnight fast at study entry. Blood plasma was separated from the blood samples, aliquoted, and stored at −80 °C without thawing until assay. Systolic and diastolic blood pressure was measured for each individual; weight and height were measured in light clothing without shoes. Body mass index (BMI) was calculated as weight (kg)/height (m)^2^. 

### 4.3. Measurement of Insulin and Adipocyte-Derived Hormones 

Plasma levels of insulin and adipocyte-derived hormones were measured as described previously [46]. Briefly, the plasma levels of adiponectin and resistin were measured by human adipokine multiplex assays (Milliplex MAP kits, EMD Millipore, Billerica, MA, USA); the levels of insulin and leptin were measured using human metabolic hormone multiplex assays (Milliplex MAP kits, EMD Millipore). All analyses were performed by the same bio-technician (T.-Y.C.). The results of these three proteins and insulin were read using a Luminex 200 system with a dynamic range of ≥3.5 log units (Luminex, Austin, TX, USA) and were collected and analyzed using an instrument equipped with MILLIPLEX Analyst software (EMD Millipore). Values for resistin and leptin are reported as ng/mL, and those of adiponectin are reported as µg/mL. Insulin levels are expressed as pg/mL. The lower limit of detection values were as follows (pg/mL): insulin (54.1), leptin (137.1), adiponectin (27.24), and resistin (5.54), respectively. The samples of leptin and insulin were assayed undiluted, and those of resistin and adiponectin were assayed diluted 1:240 in assay buffer. If the measured sample level was outside the linear range of detection, the samples were retested after an optimal dilution. All samples were measured in duplicate. The intra-assay laboratory coefficients of variation (CVs) were (%): insulin (7.2), leptin (5.2), adiponectin (6.2), and resistin (3.8). All inter-assay CVs of these four factors were <10%. Two replicates of a control sample were tested on each microplate in any given run.

### 4.4. Measurement of FSH, E2, and Biochemical Measures

Serum levels of alanine transaminase, aspartate transaminase, total cholesterol, triglycerides, low-density lipoprotein cholesterol (LDL-C), high-density lipoprotein cholesterol (HDL-C), fasting glucose, and the sex hormones E2 and FSH were measured using standard procedures at the Department of Laboratory Medicine, Changhua Christian Hospital. Briefly, the levels of FSH and E2 were measured in undiluted specimens using the Access hFSH assay and the Access Estradiol assay on the Beckman Access Immunoassay system with a dynamic range of ≥3.5 log units (Beckman Coulter, Fullerton, CA, USA). FSH levels are reported as mIU/mL and those for E2 are reported as pg/mL. The lower limit of detection was 0.2 mIU/mL for FSH and 20 pg/mL for E2, respectively. All measured specimens were analyzed in duplicate and were within the linear range of detection of the standard curve. Two duplicates of commercial control samples were tested on each microplate in any given run. The inter-assay and intra-assay laboratory CVs were <7.8% and 8.4% for E2, respectively, and they were <8.2% and 5.4% for FSH, respectively. Insulin resistance was assessed based on the homeostatic model assessment of insulin resistance (HOMA-IR) using the formula: [fasting glucose (mmol/L) × insulin (µU/L)/22.5].

### 4.5. Stastical Analysis 

Results are presented as medians (interquartile range (IQR)). The Kolmogorov-Smirnov test was used to determine whether data fit with the normal or non-normal distribution. Differences between two groups of postmenopausal women were tested by the Mann-Whitney U test, and three groups were tested by the Kruskal-Wallis test. Correlations were determined using Spearman’s rank test. The associations between FSH and adipocyte-derived hormones were determined by multivariate linear regression after logarithmic transformation for adiponectin, leptin, and LAR. The adjustment factors include the conventional risk factors BMI and age as well as the selected factors that were significant after a simple correction test. Statistical analyses were performed with SPSS software version 19.0.0 (IBM Corp., Somers, NY, USA). A two-tailed *p*-value < 0.05 was considered significant.

## 5. Conclusions

Our findings show that higher FSH levels were independently associated with circulating adiponectin levels and LAR in postmenopausal women. The results suggest that adiponectin may link FSH to metabolic relationships (e.g., insulin resistance) in postmenopausal females. Further longitudinal studies are required to clarify the causal relationships.

## Figures and Tables

**Table 1:** Characteristics of the postmenopausal participant groups.

Variables	Non-BrCa	BrCa	BrCa-Gn	*p*^a^ Value	*p*^b^ Value	*p*^c^ Value
n	261	88	59			
Age, years	54 (52, 57)	52 (47, 57)	46 (43, 49)	<0.001	<0.001	<0.001
MP_duration, years	4 (1, 8)	3 (2, 7)	2 (1, 3)	<0.001	0.924	<0.001
SBP, mmHg	115 (96, 118)	114 (95, 116)	112 (94, 109)	0.212	0.287	0.074
DBP, mmHg	71 (65, 76)	72 (63, 75)	70 (62, 73)	0.465	0.612	0.435
BMI, kg/m^2^	23.1 (21.6, 25.5)	23(21.05, 24.95)	23.9 (21.5, 26.1)	0.290	0.320	0.138
Fasting glucose, mg/dL	95 (90, 101)	93.5 (88, 101)	91 (86, 95)	0.001	0.249	0.022
Insulin, pg/mL	270.3 (149, 426)	376.5 (231, 510)	323.5 (178, 572)	0.001	<0.001	0.661
Hemoglobin A1c, %	5.5 (5.3, 5.7)	5.55 (5.2, 5.8)	5.4 (5.2, 5.6)	0.113	0.674	0.066
FSH, mIU/mL	67.9 (54, 80.4)	36.17 (26.8, 56.4)	3.19 (1.8, 5.9)	<0.001	<0.001	<0.001
Estradiol, pg/mL	20 (20, 20)	20 (20, 20)	20 (20, 20)	0.508	0.315	0.993
Total cholesterol, mg/dL	206 (186, 234)	193 (167, 216)	180 (160, 206)	<0.001	<0.001	0.054
Triglyceride, mg/dL	94.5 (67, 126)	103.5 (74, 164)	114 (79, 150)	0.007	0.010	0.882
HDL cholesterol, mg/dL	57 (49, 68)	52 (43, 64)	52 (46, 61)	0.008	0.014	0.958
LDL cholesterol, mg/dL	124.6 (104, 152)	108.4 (82, 127)	97.4 (82, 124)	<0.001	<0.001	0.392
Adiponectin, ug/mL	30.6 (16.4, 45.6)	23.7 (13.2, 39.4)	17.8 (11.5, 31.4)	<0.001	0.049	0.048
Resistin, ng/mL	22.0 (16.1, 33.7)	19.6 (16.2, 24.9)	18.6 (13.5, 28.3)	0.014	0.038	0.421
Leptin, ng/mL	7.7 (4.8, 14.1)	10.9 (6.9, 15.7)	12.7 (6.9, 22.5)	<0.001	0.003	0.222
Leptin to Adiponectin ratio	0.28 (0.14, 0.71)	0.48 (0.25, 1.05)	0.78 (0.27, 1.358)	<0.001	0.002	0.138
HOMA-IR	1.04 (0.53, 1.61)	1.39 (0.79, 2.01)	1.17 (0.61, 2.18)	0.004	0.002	0.511

Data are presented as median (Q1, Q3). Statistical analysis was conducted by Mann-Whitney U test between the two postmenopausal woman groups and by the Kruskal-Wallis test between the three postmenopausal woman groups to compare the median differences. Abbreviations: Q, quarter; Q1, 25th percentile; Q3, 75th percentile; non-BrCa, the postmenopausal women without breast cancer; BrCa, the breast cancer postmenopausal women receiving tamoxifen therapy; Gn, gonadotropin-releasing hormone analogs; MP duration, menopause period since final menstrual period; SBP, systolic blood pressure; DBP, diastolic blood pressure; FSH, follicle-stimulating hormone; BMI, body mass index; HDL, high-density lipoprotein; LDL, low-density lipoprotein; HOMA-IR, homeostatic model assessment of insulin resistance; *p*^a^ value, *p* values among non-BrCa, BrCa, and BrCa-Gn groups; *p*^b^ value, *p* values among non-BrCa, and BrCa groups; *p*^c^ values, *p* value among BrCa, and BrCa-Gn groups.

**Table 2:** Associations between FSH and adipocyte-derived hormones in the postmenopausal groups.

Variables	Beta 1 mIU/mL Increment of FSH (95% CI)	Adjusted r	*p*-Value
**Non-breast cancer women (*n* = 261)**			
Log-adiponectin	0.010 (0.002)	0.312	<0.001
Log-leptin	−0.003 (0.002)	−0.084	0.135
Log-LAR	−0.013 (0.003)	−0.265	<0.001

Adjustment for age, body mass index, and menopause duration. Abbreviations: LAR, leptin to adiponectin ratio.
